# Temperature-driven topological quantum phase transitions in a phase-change material Ge_2_Sb_2_Te_5_

**DOI:** 10.1038/srep38799

**Published:** 2016-12-13

**Authors:** S. V. Eremeev, I. P. Rusinov, P. M. Echenique, E. V. Chulkov

**Affiliations:** 1 Institute of Strength Physics and Materials Science, 634055, Tomsk, Russia; 2 Tomsk State University, 634050 Tomsk, Russia; 3 Saint Petersburg State University, Saint Petersburg, 198504, Russia; 4 Donostia International Physics Center (DIPC), 20018 San Sebastián/Donostia, Basque Country, Spain; 5 Departamento de Física de Materiales UPV/EHU, Centro de Física de Materiales CFM - MPC and Centro Mixto CSIC-UPV/EHU, 20080 San Sebastián/Donostia, Basque Country, Spain

## Abstract

The Ge_2_Sb_2_Te_5_ is a phase-change material widely used in optical memory devices and is a leading candidate for next generation non-volatile random access memory devices which are key elements of various electronics and portable systems. Despite the compound is under intense investigation its electronic structure is currently not fully understood. The present work sheds new light on the electronic structure of the Ge_2_Sb_2_Te_5_ crystalline phases. We demonstrate by predicting from first-principles calculations that stable crystal structures of Ge_2_Sb_2_Te_5_ possess different topological quantum phases: a topological insulator phase is realized in low-temperature structure and Weyl semimetal phase is a characteristic of the high-temperature structure. Since the structural phase transitions are caused by the temperature the switching between different topologically non-trivial phases can be driven by variation of the temperature. The obtained results reveal the rich physics of the Ge_2_Sb_2_Te_5_ compound and open previously unexplored possibility for spintronics applications of this material, substantially expanding its application potential.

The Ge_2_Sb_2_Te_5_ compound is a phase-change material (PCM) which has been long used in optical memory devices such as re-writable DVD-RAM and is also a leading candidate for next generation non-volatile electronic memory known as phase-change random-access memory (PC-RAM)^1^,^2^. Recently, a new concept of nanostructured PCMs has been developed based on [GeTe]_*n*_[Sb_2_Te_3_]_*m*_ short-period superlattices, referred to as interfacial phase-change materials^3^,^4^,^5^ among which Ge_2_Sb_2_Te_5_ (*n* = 2, *m* = 1) is regarded as a prototype conventional PCM. Initially it was assumed that in this type of materials the switching between memory states is due to the amorphous-crystalline phase-transition of the separate relatively thick superlattice sublayers^4^,^5^,^6^. However, latter it was demonstrated that the superlattice kept functioning while the GeTe sublayer thickness was narrowed down to 2–3 GeTe bilayers and thus it was concluded that the temperature-induced phase-change occurred within the crystalline state, rather than between amorphous and crystalline phases, as was verified with transmission electron microscopy^3^.

At present, it is established that Ge_2_Sb_2_Te_5_, i.e. [GeTe]_*n*=2_[Sb_2_Te_3_]_*m*=1_ system can adopt four different hexagonal layered structures in which the primary bonds in different layers are aligned according to the ordering of Ge, Sb and Te layers^7^. They are Kooi structure, experimentally the most stable phase of Ge_2_Sb_2_Te_5_^8^, which is formed by nonuple layer (NL) building blocks, where GeTe bilayers are incorporated into the Sb_2_Te_3_ quintuple layer (QL); Petrov structure^9^, in which the [GeTe]_2_ blocks are sandwiched between Sb_2_Te_3_ QLs with Ge-Te-Te-Ge sequence within the block; Inverted-Petrov structure with Te-Ge-Ge-Te sequence; and so-called Ferro structure, in which the atomic layer sequence in the [GeTe]_2_ block has Ge-Te-Ge-Te order, i.e. like in ferroelectric bulk GeTe. The relative stability of these four structures depends on temperature. Earlier it was shown by means of *ab-initio* calculations of enthalpy as a function of temperature^10^ the Kooi phase has the lowest enthalpy at 0 K, in agreement with earlier density functional theory (DFT) calculation^7^ and experiment^8^. However, upon raising the temperature the enthalpy of the Kooi structure increases and above ≈125 K the Ferro phase becomes the most stable one^10^. The metastable Petrov and Inverted Petrov phases are involved into atomic mechanism of the phase transition between stable Kooi and Ferro phases^10^. Despite the results of ref. 10 correctly reproduce the sequence and stability of the Ge_2_Sb_2_Te_5_ crystal structures they underestimate the transition temperature which according to experiments is higher than the room temperature.

Despite the advances in comprehension of the crystal structure and atomic mechanisms of crystal phase transformations the electronic structure of Ge_2_Sb_2_Te_5_ phases is currently not fully understood. For the stable low-temperature NL structured Kooi phase the narrow-gap band insulator (BI) phase has been obtained theoretically^7^,^11^,^12^,^13^. It is in contrast to related NL-structured Ge_2_Bi_2_Te_5_ and Sn_2_Sb(Bi)_2_Te_5_ compounds, which are shown to be topological insulators (TIs)^12^,^14^. The [GeTe]_*n*_[Sb_2_Te_3_]_*m*_ compound with opposite *n, m* indices (*n* = 1, *m* = 2) has also been predicted to be TI^15^. The calculations for Ferro and Petrov structures also predicted the BI phase, while another metastable structure, the Inverted Petrov structure is shown to possess the Dirac semimetal quantum phase^7^.

In this work, we focus on electronic structure of stable low-temperature Kooi and high-temperature Ferro structures. We start with ordered Kooi structure which was also considered in the previous works^7^,^12^. The equilibrium crystal structure of the Kooi phase, obtained using VASP code (see details in the Method section) is shown in Fig. 1(a). The calculated bulk band structure demonstrates the insulating state with a gap of 25.2 meV in the middle of the Γ-A direction (Fig. 1(b), red lines) which is trivial band insulator in terms of the 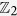 topological invariant that is in agreement with earlier results^7^,^11^,^12^.

The obtained small band-gap value may be an indication that the system is close to the topological quantum phase transition (TQPT) and can be converted into the topological phase by increasing spin-orbit interaction strength. We artificially increased the spin-orbit interaction strength *λ* in the ordered equilibrium structure and found that it leads to shift of the gap towards the A point along with its narrowing. Upon further increasing the spin-orbit interaction strength the system has gone through the critical point of the TQPT (at *λ/λ*_0_ ≈ 1.2), the gap becomes inverted achieving at *λ/λ*_0_ = 1.4 a width of 76 meV at the A point (Fig. 1(c)). This result is in line with the fact that the TI phase was predicted for Ge_2_Bi_2_Te_5_^12^ in which the atomic spin-orbit coupling strength is larger owing to the larger atomic mass of Bi as compared to Sb. The bulk band inverted topology should manifest itself in formation of the spin-polarized Dirac state at the surface. As can be seen in Fig. 1(d) the gapless surface state with typical for related NL-structured Ge_2_Bi_2_Te_5_ and Sn_2_Sb(Bi)_2_Te_5_ TIs dog-leg dispersion^12^,^14^ arises.

However, it is known from experiments that stable Ge_2_Sb_2_Te_5_ contains mixed Ge/Sb atomic layers^16^. Earlier, the influence of Ge/Sb mixing on electronic structure of the Ge_2_Sb_2_Te_5_ Kooi phase was considered within ordered 2 × 2 supercell approach^11^. It was demonstrated that 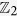 invariant depends on relative concentration of Ge in the inner and outer Ge/Sb layers of NL (which was varied from 0 to 100% in steps of 25% in that model, containing 4 atoms in an atomic plane). It was shown that the material is trivial BI when the Ge atoms completely occupy inner layers (as in Fig. 1(a)) while it is TI at 50/50 mixing in the Ge/Sb layers.

We took into account the Ge/Sb mixing in the calculations within virtual crystal approximation (VCA) using ABINIT code. First we checked the band structure for the ordered phase with ABINIT code and found it in a good agreement with the VASP result (Fig. 1(b), dashed blue lines). The calculated spectrum has a gap of 29.5 meV in the middle of the Γ-A direction. Next, according to the experiment^16^, we constructed Ge_0.56_/Sb_0.44_ and Ge_0.44_/Sb_0.56_ virtual atoms for the outer and inner Ge/Sb layers of NL, respectively. We find that at normal, zero-pressure conditions the minimum gap of 61 meV appears at the A point (Fig. 1(e)) and, being inverted (Fig. 1(f)) results in non-trivial topological invariant 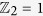. This signifies that the Kooi structure possesses the TI quantum phase. Thereby we can conclude that Ge/Sb mixing in the Ge_2_Sb_2_Te_5_ Kooi structure effectively increases the spin-orbit interaction. As can be seen in Fig. 1(g) presenting the surface band structure, calculated for 5 NL slab of the Kooi phase with Ge/Sb mixing, the topological surface state with the Dirac point at the Fermi level arises in the spectrum.

The distinctive feature of the Ferro structure as opposed to other Ge_2_Sb_2_Te_5_ phases is the lack of inversion symmetry owing to Ge-Te-Ge-Te layer sequence in the [GeTe]_2_ block (Fig. 2 (a)). It results in the spin-orbit splitting of the bulk energy spectrum (Fig. 2(b)) which resembles the Rashba-like band splitting in the bulk GeTe^17^. The presented spectrum has a tiny gap of 11 meV in the A–H direction at *k*_*x*_ ≈ 0.1 Å^−1^ away from the A point. Near the gap the spin texture differs from Rashba spin-helical picture and demonstrates almost collinear spin alignment perpendicular to the A–H (*k*_*x*_) direction with sizable *S*_*z*_ component of the same sign both below and above the gap (Fig. 2(c)). Away from the H–A–L plane at *k*_*z*_ = *π/c* ± 0.015 Å^−1^ (where *k*_*z*_ = *π/c* is the H–A–L plane) the gap closes forming a pair of the Weyl nodes (Fig. 2(d)) that is distinctive feature of the topological Weyl semimetal (TWS) phase.

In principle, TWS can be realized by breaking either time-reversal or inversion symmetry of topological Dirac semimetals^18^. In this regard, the existence of the TWS in the Ferro phase can be understood from the comparison with the intermediate Inverted Petrov structure. The Inverted Petrov structure possessing the Dirac semimetal phase differs from the Ferro structure only in the atomic layer sequence within [GeTe]_2_ block so that the latter one is inversion asymmetric.

The Weyl fermions in the bulk are predicted to provide realization of the chiral anomaly, giving rise to a negative magnetoresistance under parallel electric and magnetic fields, the semi-quantized anomalous Hall effect, unusual optical conductivity, non-local transport and local non-conservation of the current^19^,^20^,^21^,^22^,^23^,^24^,^25^. At the surface the bulk band topology should manifest itself via formation of unusual surface states which form disjoint Fermi arcs which connect the projections of the pairs of Weyl nodes onto the surface Brillouin zone^26^,^27^. The Fermi arc surface states are predicted to show unconventional quantum oscillations in magneto-transport, as well as unusual quantum interference effects in tunneling spectroscopy^27^,^28^,^29^,^30^.

The Ferro phase can adopt six different surface terminations depending on Sb_2_Te_3_ QL and GeTe bilayers sequence near the cleavage plane. These terminations should differ in bending of the surface potential owing to polarity in the GeTe bilayers (Ge^+0.4^, Te^−0.4^). The geometries of the terminations are shown schematically in insets in Fig. 3. The calculated surface electronic spectra (Fig. 3, odd columns) demonstrate that all surface terminations hold the trivial Rashba-split surface states resulting from splitting off from bulk bands due to the band-bending effect which is negative for QL-GeTe-GeTe-, GeTe-GeTe-QL-, and GeTe-QL-GeTe- terminations (first column in Fig. 3) and positive for terminations shown in the third column. Besides these states, as can be seen in the Fermi surface maps (Fig. 3, even columns), each surface termination holds the Fermi arcs connecting the Weyl’s pairs. In most cases they connect the Weyl nodes within each 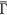-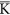 pair while in case of TeGe-QL-TeGe- termination the arcs connect points of neighboring pairs via the hole-like Rashba surface state which crosses the Fermi level twice in the 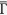-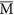 direction, however does it once in 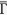-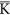 and touches the Weyl nodes on the conduction band side. Similar effect of the Weyl nodes reconnecting has been observed recently in the TWS phase arising in BiTeI under pressure^31^.

In summary, on the basis of *ab-initio* calculations we provide an important ingredient to the physics of prospective phase-change material Ge_2_Sb_2_Te_5_ demonstrating that temperature-induced structural phase transformation is accompanied by the quantum topological phase transition from TI phase in the low-temperature Kooi crystal structure to TWS phase in the high-temperature Ferro structure. We also demonstrate that the Ge/Sb mixing in the low-temperature structure is crucial for formation of the TI phase. Together with earlier predicted Dirac semimetal phase for intermediate metastable Inverted Petrov structure the TI and Weyl semimetal phases form a rich topological family realized in the same material and switching between the topological phases is ensured by the temperature. The Dirac surface states of TI, prospective for spintronic applications as well as exotic bulk and surface electronic states of TWS providing an ideal platform for many novel physical phenomena, such as negative magnetoresistance, anomalous quantum Hall effect and chiral magnetic effect can be realized in Ge_2_Sb_2_Te_5_, substantially expanding the application potential of this material. Since Ge_2_Sb_2_Te_5_ compound naturally has a polycrystal structure with randomly oriented crystallites the utilization of the predicted surface states of the topological quantum phases requires precise epitaxial growth of the Ge_2_Sb_2_Te_5_ films.

## Methods

Electronic structure calculations were carried out within density functional theory (DFT). For bulk band structure calculations we used the Vienna Ab Initio Simulation Package (VASP)^32^,^33^. The interaction between the ion cores and valence electrons was described by the projector augmented-wave (PAW) method^34^,^35^. Relativistic effects, including spin-orbit interaction (SOI), were taken into account. For this calculations, the PBE exchange-correlation functional^36^ was used and DFT-D3 van der Walls correction^37^ was applied for accurate structure optimization. To treat the disordered Kooi phase we employed a virtual crystal approximation (VCA) as implemented in the ABINIT code^38^, where the averaged potential of a virtual atom occupying a site in the Ge/Sb sublattice is defined as a mixture *V*_V*CA*_ = *xV*_G*e*_ + (1 − *x)V*_S*b*_ of Ge (*V*_G*e*_) and Sb (*V*_S*b*_) pseudopotentials. In ABINIT calculations we used GGA-PBE Hartwigsen-Goedecker-Hutter (HGH) relativistic norm-conserving pseudopotentials which include the SOI^39^. For surface electronic structure calculations for the Weyl phase, first the results of VASP calculations were used in the WANNIER90 code^40^ to construct tight-binding model. The chosen basis consists of six spinor *p*-type orbitals for each atom: 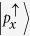, 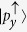, 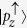, 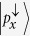, 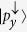, 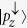. The low-lying *s* orbitals are not taken into consideration. Surface tight-binding model is derived from the bulk one with inclusion of band-bending effects obtained from direct surface calculations within DFT. The surface spectrum has been calculated within surface Green function approach^41^,^42^. The 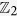 invariant is calculated from the parity of occupied electronic states at the time-reversal invariant points of the bulk Brillouin zone^43^.

## Additional Information

**How to cite this article**: Eremeev, S. V. *et al*. Temperature-driven topological quantum phase transitions in a phase-change material Ge_2_Sb_2_Te_5_. *Sci. Rep.* **6**, 38799; doi: 10.1038/srep38799 (2016).

**Publisher's note:** Springer Nature remains neutral with regard to jurisdictional claims in published maps and institutional affiliations.

## Figures and Tables

**Figure 1:**
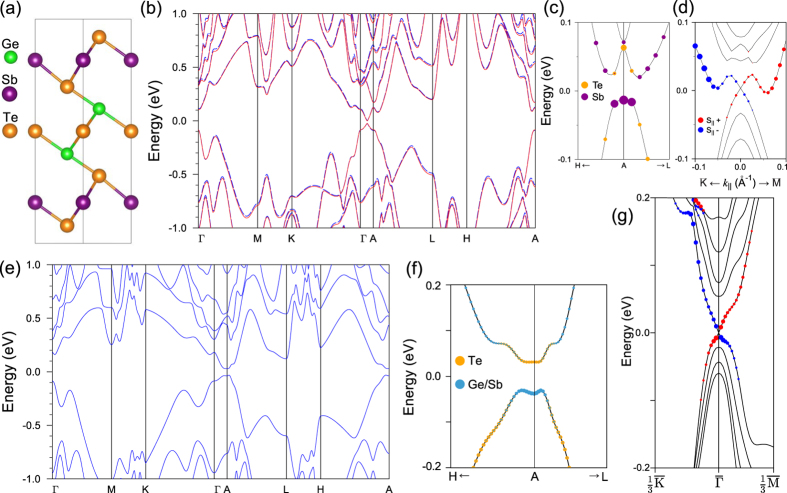
(**a**) Unit cell of the low-temperature Ge_2_Sb_2_Te_5_ Kooi phase. (**b**) Bulk band structure of ordered Kooi phase with equilibrium crystal structure parameters (red solid lines – VASP calculation, blue dashed lines – ABINIT calculation). Electronic structure of the ordered Kooi structure with *λ/λ*_0_ = 1.4: (**c**) bulk band structure near the A-gap with indication of the weights of Te and Sb orbitals, (**d**) surface electronic structure. (**e**) Bulk band structure of Kooi-Ge_2_Sb_2_Te_5_ with Ge/Sb mixing, and (**f**) its magnified view near the A-gap with indication of the weights of Te and Ge/Sb *p*_*z*_ orbitals (VCA-ABINIT calculation). (**f**) Surface electronic spectrum for Kooi-Ge_2_Sb_2_Te_5_ with Ge/Sb mixing. Red and blue circles demote the positive and negative sign of the in-plane spin, respectively.

**Figure 2:**
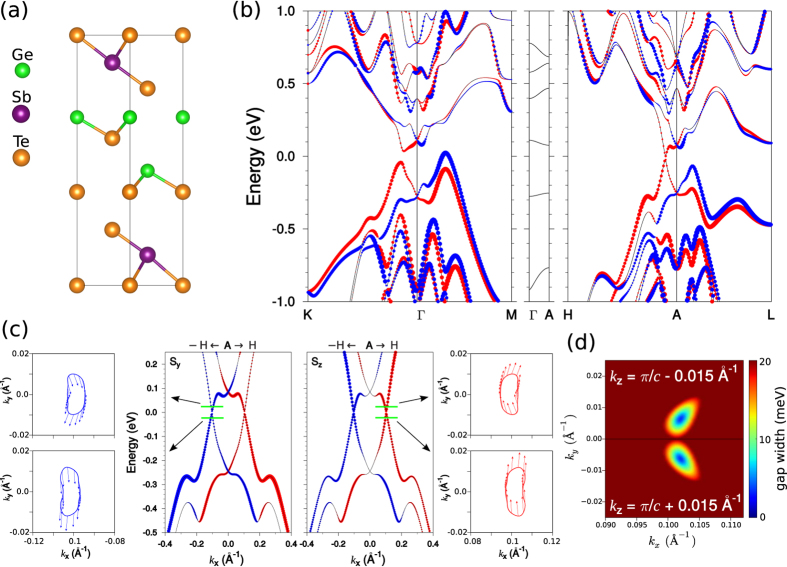
(**a**) Unit cell of the high-temperature Ferro-Ge_2_Sb_2_Te_5_ structure. (**b**) Bulk band spectrum; red/blue circles indicate positive/negative sign of in-plane, *S*_*xy*_ spin components. (**c**) Magnified view of the spectrum in the vicinity of the A point in -H–A–H direction with indication of *S*_*y*_ and *S*_*z*_ spin components (middle panels) and spin-resolved constant energy contours taken below and above the gap (left and right panels for -H–A and A–H directions, respectively); here arrows show in-plane spin direction and red/blue color correspond to positive/negative sign of *S*_*z*_. (**d**) The dependence of the gap width on *k*_||_ at *k*_*z*_ = *π/c* − 0.015 and *k*_*z*_ = *π/c* + 0.015 Å^−1^; the zero-gap points correspond to the Weyl nodes.

**Figure 3:**
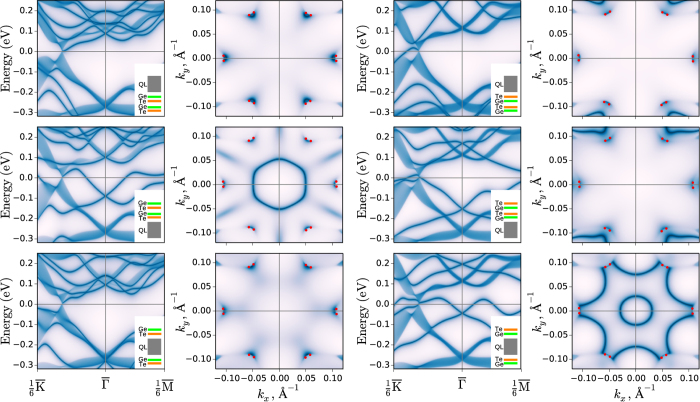
Surface electronic structure for different Ferro-Ge_2_Sb_2_Te_5_ surface terminations (odd columns) which structure is shown in insets and corresponding Fermi surfaces (even columns). Small red points on Fermi surfaces mark positions of the Weyl nodes. *k*_*x*_ and *k*_*y*_ axis directed along 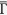-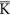 and 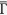-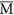, respectively.
